# Adult attachment and love psychological stress among college students: the mediating role of core self-evaluation and the moderating role of meaning in life

**DOI:** 10.1186/s40359-024-01990-5

**Published:** 2024-09-10

**Authors:** Minghua Song, Xianman Hu, Shen Liu

**Affiliations:** 1 Mental Health Education Guidance Center, Huzhou University, Huzhou, Zhejiang China; 2 Department of Information Management, Anhui Vocational College of Police Officers, Hefei, 230036 Anhui China; 3 Department of Psychology, School of Humanities and Social Sciences, Anhui Agricultural University, Hefei, 230036 Anhui China

**Keywords:** Adult attachment; love psychological stressors, Core self-evaluation, Meaning in life, College students

## Abstract

**Background:**

With college students going into dating relationships, dating partners become their new attachment figures. We aimed to investigate the relationship between adult attachment, and love psychological stress among college students, which also explored the roles of core self-evaluation and meaning in life.

**Methods:**

We conducted a questionnaire survey on 813 college students using the adult attachment scale, core self-evaluation scale, meaning in life scale, and love psychological stress scale. We constructed a moderated mediation model to analyze the relationship between adult attachment and love psychological stress, as well as the mediating effect of core self-evaluation and the moderating effect of meaning in life.

**Results:**

The results showed that after controlling for single parent or not, adult attachment significantly negatively predicted love psychological stress of college students. Core self-evaluation partially mediated the relationship between adult attachment and love psychological stress of college students. The second half of the mediation model was moderated by meaning in life, that is, with the increase of meaning in life, the negative predictive effect of core self-evaluation on love psychological stress of college students gradually strengthened. The findings of this study demonstrate the detrimental impact of adult attachment on love psychological stress of college students, as well as the mediating core self-evaluation and the moderating role of meaning in life.

**Conclusions:**

The mediating and moderating effect of adult attachment between love psychological stress, as well as the mediating effect of core self-evaluation and the moderating effect of meaning in life were confirmed. Overall, promoting the healthy development of adult attachment and helping them shape a positive meaning in life can enhance individuals’ core self-evaluation, thus alleviating love psychological stress among college students. It can also provide references for mental health education in colleges and universities.

## Introduction

The college student population is in the early stages of adulthood, which is a crucial stage for seeking and establishing intimate relationships [1]. During this stage, individuals are faced with a series of life transitions such as entering romantic relationships, leading to emotional fluctuations and psychological changes, thus causing college students to experience love psychological stress [2]. In recent years, concerns about college students’ love psychology have been increasing, and it has also received more and more attention from researchers [3, 4]. Research has shown that the quality of college students’ love relationships can affect their mental health [5]. If not handled properly, individuals may experience or perceive significant stress, which can affect their physical and mental health, such as insomnia, anxiety, depression, and at severe levels, even increasing the risk of suicide [6]. Therefore, this study will delve into the mechanisms of the emergence and alleviation of love psychological stress among college students, which is of great significance for promoting their physical and mental health.

According to the theory of stress cognitive evaluation, an individual’s attachment system conducts primary and secondary assessments in the face of stress, which in turn affects their level of stress perception [7]. Attachment, as an inherent mechanism, runs through the entire life cycle of an individual and plays a significant role in their development, including psychological stress, social function, mental health, etc. [8, 9]. Adult attachment, an important extension of early parent-child attachment, is a more enduring emotional connection between individuals and their current intimate partners or peers, which can affect their interpersonal function and social life [10]. Individuals with secure attachment tend to actively communicate and interact with others, share emotions with each other, and are more likely to experience positive emotions [11], whereas individuals with insecure attachment (including attachment avoidance and anxiety) tend to close off their emotions, have less communication with others, and experience negative emotions more easily [4]. Individual differences in adult attachment are closely related to the outcome of intimate relationships. For example, individuals with insecure attachment styles often find it more difficult to have good romantic relationships and are more prone to experiencing anxiety and depressive emotions [12], which can easily lead to love psychological stress. From this, it can be inferred that the formation and development of adult attachment styles can influence the love psychological stress of college students to a certain extent. Based on this, the study proposes hypothesis 1: Adult attachment significantly and positively predicts the love psychological stress among college students (*H*_1_).

The internal working model of attachment states that the process of interaction between children and their nurturers in early childhood helps them form mental representations or cognitive schemas about themselves, which leads to the formation of different attachment types. The attachment patterns formed in childhood are relatively stable and influence the emotional and behavioral styles of individuals in adulthood. Individuals with insecure attachment tend to attribute themselves inward in the face of negative events, seeking “their own faults” and preemptively deny their self-worth, thus posing a threat to their self-esteem and leading to low self-esteem [13]. Core self-evaluation, as an important psychological cognitive resource, is the most basic evaluation of individuals’ own abilities and values, including traits such as self-esteem, general self-efficacy, neuroticism, and locus of control [14]. Research has found that insecure attachment significantly negatively predicts self-esteem [14], and self-esteem is one of the important traits that make up core self-evaluation, which means that insecure attachment and core self-evaluation have a close connection. It has also been shown that individuals with higher levels of attachment avoidance and attachment anxiety hold negative evaluations of others as well as themselves, and also have difficulty establishing good interpersonal relationships [11]. In addition, compared to individuals with higher self-evaluation, individuals with lower self-evaluation have more negative cognitive schemas, are more sensitive to negative information, and are more negative and passive in seeking intimate relationships [14], which can lead to love psychological stress. It is inferred that adult attachment will indirectly form love psychological stress by influencing core self-evaluation of college students. Based on this, the present study proposed hypothesis 2: Core self-evaluation plays a mediating role between adult attachment and college students’ love psychological stress (*H*_2_).

Meaning in life refers to individuals’ realization and understanding of the meaning of their life and awareness of their goals, tasks, or missions in life [15]. Meaning in life can be categorized into seeking a sense of meaning in life and possessing a sense of meaning in life. Seeking a sense of meaning in life and possessing a sense of meaning in life are relatively independent, and individuals who seek a sense of meaning in life does not necessarily have a sense of meaning in life [15, 16]. The meaning construction model suggests that acquiring a sense of meaning is a complex process that involves comparing and evaluating the sense of meaning gained from situational stimuli with one’s existing beliefs and overall sense of meaning. If the assessment of situational sense of meaning is consistent with the general sense of meaning assessment, the individual’s sense of meaning will be successfully adapted and new meaning will be obtained. Conversely, if there is a discrepancy between the two assessments, it will bring great stress to the individual [16]. Individual characteristics (sense of meaning) can moderate the relationship between environmental factors (adult attachment) and personal psychological or behavioral aspects (core self-evaluation and stress). Individuals with the same level of adult attachment who have a stronger meaning in life tend to have higher core self-evaluation. Similarly, with consistent levels of adult attachment, the stronger the meaning in life, the less the love psychological stress. Recent research has found that the meaning in life is closely related to core self-evaluation and stress [17, 18]. Studies have shown that individuals with a lower meaning in life are prone to feelings of emptiness and helplessness, exhibiting more depressive and anxious emotions [19], and being more negative and passive in seeking intimate relationships [14], thus experiencing love psychological stress. Therefore, this study proposes hypothesis 3a: meaning in life moderates the influence of adult attachment on the love psychological stress of college students (*H*_3a_). Additionally, research has shown that individuals with a well-developed meaning in life experience more positive emotions and promote self-awareness, improving the level of core self-evaluation [20]. Thus, hypothesis 3b is proposed: meaning in life moderates the first half of the mediating process of adult attachment, core self-evaluation, and love psychological stress among college students (*H*_3b_). Based on the goal process theory, negative attributions, hopelessness perceptions, and self-denial will occur when individuals do not achieve their desired goals. This means that individuals with insufficient development of life meaning will experience more negative emotions [21], thereby reducing their core self-evaluation level and exhibiting more negative and passive behavior in seeking intimate relationships [14], leading to psychological pressure in love. From this, it can be inferred that regardless of the type of internal working model of attachment faced, individuals with high meaning in life may experience more positive emotions, improve their core self-evaluation level, and be more active and proactive in intimate relationships, thus exhibiting less psychological stress in love. Therefore, this study proposes hypothesis 3c: meaning in life moderates the latter half of the mediating process of adult attachment, core self-evaluation, and love psychological stress among college students (*H*_3c_).

**Fig. 1:**
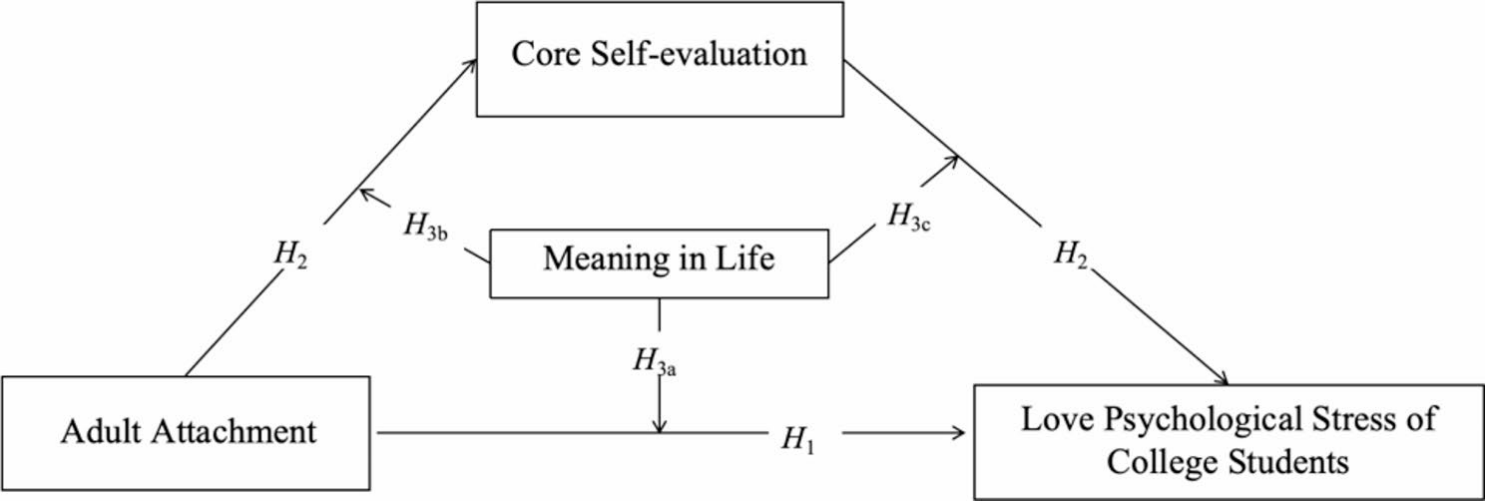
Moderated mediation model

In summary, this study primarily uses the cognitive appraisal theory of stress and the goal progress theory as theoretical foundations to explore the impact of adult attachment on the love psychological stress of college students. It further examines whether core self-evaluation plays a mediating role and whether meaning in life plays a moderating role in this process. The theoretical model illustrating the relationships between the variables can be found in Fig. 1.

## Methods

### Study design and participants

An a priori power analysis revealed that to test a simple mediation model with an anticipated small effect size for the view between adult attachment to core self-evaluation and between core self-evaluation to love psychological stress indicated a minimum of approximately 450 participants would be needed to test for mediation using the parametric bootstrap procedure for calculating the standard error of the indirect effect [22]. In order to adequately power a test of moderated mediation, we anticipated needing a minimum sample size of 400 participants (using bootstrapped procedure to estimate SE) for potential moderated mediation given a small effect size [23]. We selected students from seven universities in Anhui and Zhejiang, China. We chose these seven universities because they presented rural, industrial, and economic territories. 850 college students were recruited via questionnaires and scales. Among them, 37 college students were excluded owing to missing or consecutive answers the same option. Finally, 813 valid data points were obtained with an effective recovery rate of 95.65%. Among the 813 participants, 178 were male and 635 were female between 18 and 24 years old, with an average age of 19.87 years (*SD* = 1.33). In addition, there were 475 freshmen, 177 sophomores, 126 juniors, and 35 seniors. And 87 were single parents, and 726 were non-single parents. All subjects participated in the study voluntarily. After completing the study, the subjects received a signing pen (about 5 RMB) as a token of appreciation. All subjects gave written informed consent in accordance with the ethical principles of the Declaration of Helsinki.

### Measures

#### Adult attachment scale

The revised edition of adult attachment scale (REA), developed by Collins et al. [13]and revised by Wu et al. [24], was used to assess adult attachment styles. The scale consists of 18 items, including the dimensions of closeness, dependence, and anxiety. The closeness and dependence dimensions are highly correlated, and the closeness and dependence dimensions were merged to produce a composite closeness-dependence dimension, with sample items such as “I find that people do not want to be as close to me as I would like them to be”. A 5-point Likert scale was used, with “not at all consistent” scoring 1 point and “completely consistent” scoring 5 points. In this study, only the closeness and dependence subscales were used, and the total mean score of the items was calculated, with higher scores indicating a greater degree of attachment. The internal consistency coefficient for this scale in this study was 0.85.

#### Core self-evaluation scale

The core self-evaluation scale, developed by Judge et al. [25] and revised by Du et al. [26], was used to understand the most basic evaluations that individuals hold about their own abilities and values. The scale consists of 10 items, with a single dimension. An example items such as “I feel bad and hopeless about many things”. A 5-point Likert scale was used, with “completely disagree” scoring 1 point and “completely agree” scoring 5 points. The total mean score for all items was calculated, with higher scores indicating higher core self-evaluation. In this study, the internal consistency coefficient of the scale was 0.90.

#### Mean in life scale

The Chinese meaning in life scale, developed by Steger et al. [27] and revised by Wang and Dai [28], is used to measure an individual’s meaning in life. The scale consists of 10 items, covering two dimensions: the experience of meaning in life and the search for meaning in life. An example item is “I am looking for something that makes my life meaningful.” It uses a 7-point Likert scale, where “completely disagree” is scored as 1 point and “completely agree” is scored as 7 points. The overall average score of all items is calculated, with higher scores indicating a stronger sense of meaning in life. In this study, the internal consistency coefficient of the questionnaire is 0.88.

### Love psychological stress scale

The psychological stressor scale of college students’ love, developed by Zhang [29], was used to understand the psychological stress in college students’ love relationship and their overall feelings of stress. The scale consists of 27 items, including six dimensions: ability-related, family influence, personality traits, reciprocal feelings (psychosexual), value tendency, and academic burden, with sample items such as “I am worried that our views on love and values are different”. A 5-point Likert scale was used, with “none” scoring 1 and “often” scoring 5. The weighted mean score of all items was calculated, with higher scores indicating greater psychological stress in relationships. In this study, the internal consistency coefficient of the scale was 0.96.

### Procedure

After obtaining the informed consent of the students themselves, this study was conducted a collective survey on a class basis. The participants filled out the questionnaires in a quiet classroom, with trained psychology students as the subjects. Before conducting the survey, the subjects explained the requirements, the instructions, and provided timely guidance when the participant encountered problems. The participants anonymously filled out the questionnaires, collected it on the spot for numbering (approximately 20 min). After the end, the subjects explained the research purpose to the participants and asked if they have guessed the research purpose.

### Data analysis

Descriptive statistics and correlation analyses of the main variables were conducted using SPSS 26.0. Model 15 (http://www.Afhayes.com/download) in the PROCESS programme plug-in developed by Hayes [30] was used to test for specific mediating and moderating effects.

## Results

### Common method biases test

All the questionnaires in this study were completed anonymously, so the Harman single-factor test was used to test the common method bias [46]. The results showed that there were ten factors with eigenvalues greater than 1, and the cumulative variation explained by the first factor accounted for only 20.13%, less than 40%, so there was no serious common method bias.

### Correlation analysis of each variable

The correlations of the variables in this study are shown in Table 1. Adult attachment is significantly positively correlated with core self-evaluation and meaning in life, but significantly negatively correlated with love psychological stress; core self-evaluation is significantly positively correlated with meaning in life and significantly negatively correlated with love psychological stress; and meaning in life is significantly negatively correlated with love psychological stress. This suggests that the data obtained in this study are suitable for subsequent analysis. In addition, since single parent or not was significantly correlated with core self-evaluation and meaning in life, it was used as a control variable in the subsequent study.

**Table 1:** Descriptive statistics and correlation analysis results of each variable

Variables	M	SD	1	2	3	4	5
1 Single parent or not	—	—	—				
2. Adult attachment	3.28	0.49	0.06	—			
3. Core self-evaluation	2.85	0.67	0.08^*^	0.54^***^	—		
4. Meaning in life	5.05	0.95	0.11^**^	0.32^***^	0.47^***^	—	
5. Love psychological stress	2.38	0.80	-0.06	-0.42^***^	-0.45^***^	-0.24^***^	—

Note: ^*^p < 0.05, ^**^p < 0.01, ^***^p < 0.001. The same below

### The mediating role of core self-evaluation

Firstly, standardize all variables and test the mediating effect of core self-evaluation. The results showed (see Table 2) that adult attachment significantly positively predicted core self-evaluation (β = 0.73, *p* < 0.001) and significantly negatively predicted love psychological stress (β=-0.41, *p <* 0.001), and that core self-evaluation significantly negatively predicted love psychological stress (β=-0.38, *p <* 0.001). A bias-corrected percentile Bootstrap-based method found that core self-evaluation partially mediated the relationship between adult attachment and college students’ love psychological stress, the mediating effect was − 0.28, and the 95% confidence interval was [-0.36, -0.19], which accounted for 41.18% of the total effect.

**Table 2:** Mediation model test of core self-evaluation

Predictive Variables	Model 1: Love Psychological Stress		Model 2: Core Self-evaluation		Model 3: Love Psychological Stress	
	β	t	β	t	β	t
Single parent or not	-0.10	-1.19	0.10	1.64	-0.06	-0.74
Adult attachment	-0.68	-13.16^***^	0.73	18.21^***^	-0.41	-6.91^***^
Core self-evaluation					-0.38	-8.67^***^
*R2*	0.18		0.30		0.25	
*F*	88.59^***^		169.60^***^		89.52^***^	

Note: Single parent or not is a dummy variable, single parent = 1, non-single parent = 2. The same below

### Moderated mediation model test

Moderated effect analysis after standardization of all core variables showed (see Table 3) that adult attachment significantly and positively predicted core self-evaluation in Eq. 1 (β = 0.73, *p <* 0.001). In Eq. 2, the product term of core self-evaluation and meaning in life significantly and negatively predicted love psychological stress (β=-0.10, *p* = 0.003). Adult attachment, core self-evaluation, meaning in life, and love psychological stress constituted a moderated mediation effect model.

**Table 3:** Moderated mediation model test

Predictive Variables	Model 1: Core Self-evaluation			Model 2: Love Psychological Stress		
	β	t	95%CI	β	t	95%CI
Single parent or not	0.10	1.64	[-0.02, 0.23]	-0.07	-0.94	[-0.23, 0.08]
Adult attachment	0.73	18.21^***^	[0.65, 0.80]	-0.40	-6.76^***^	[-0.52, -0.28]
Core self-evaluation				-0.35	-7.41^***^	[-0.44, -0.26]
Meaning in Life				-0.03	-0.85	[-0.08, 0.03]
Core self-evaluation×Meaning in Life				-0.10	-3.02^**^	[-0.17, -0.04]
*R2*	0.30			0.24		
*F*	169.57^***^			43.03^***^		

**Fig. 2:**
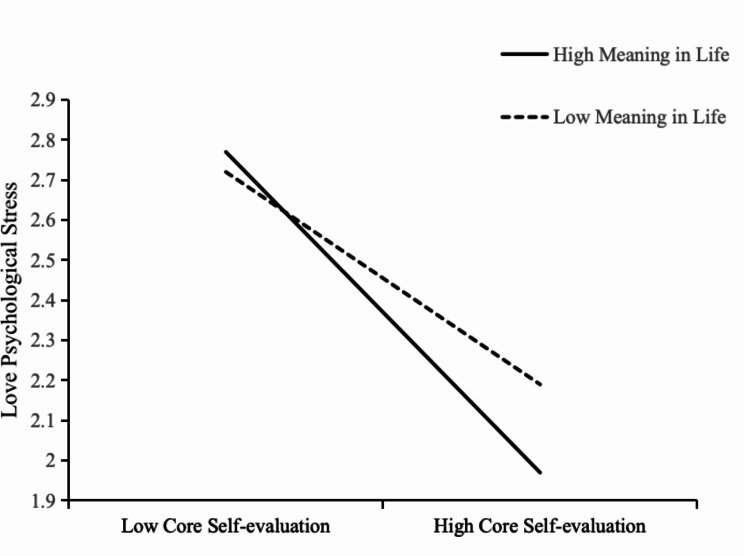
Moderating role of meaning in life in the relationship between core self-evaluation and love psychological stress

In order to explain the moderated mediation model more clearly, the meaning in life was divided into two groups according to plus or minus one standard deviation into high (*M* + 1*SD*) and low (*M-*1*SD*) for the simple slope test. The results showed (see Fig. 2) that core self-evaluation negatively predicted love psychological stress significantly when meaning in life was low (*B*simple=-0.40, *t*=-6.74, *p* < 0.001), and that core self-evaluation negatively predicted love psychological stress remained significant when meaning in life was high (*B*simple=-0.60, *t*=-12.22, *p* < 0.001). That is, individuals with a high meaning in life were able to generate less psychological stress in love at the same level of core self-evaluation relative to individuals with a low meaning in life.

## Discussion

The result of this study showed that adult attachment had a positive predictive effect on the love psychological stress of college students, that is, individuals with less secure attachment had higher levels of love psychological stress and more pronounced stress responses. Hypothesis 1 was thus validated. This result is to some extent consistent with previous research findings [31, 32]. Attachment can provide individuals with a safe base and is also an important source of their sense of security. individuals with insecure attachment experience lower levels of security. Low sense of security leads to individuals feeling neglected and not accepted, accompanied by strong hostility and inferiority complex, which makes them more prone to negative emotions such as depression and anxiety [11]. Individuals with insecure attachment have negative self-representations and positive representations of others. This makes them have low evaluations of themselves and high evaluations of others, thus craving recognition and support from others, afraid of being rejected and abandoned by others, and trying to hide their true thoughts in order to portray the image that others like [9]. Therefore, individuals with insecure attachment have a lower tendency to seek social support and have a lower belief in receiving support in stressful situations, making it difficult for them to actively face problems and experience more psychological stress. This suggests that parents should pay attention to the formation of early safety attachment in their children, provide them with sufficient love and support, create a warm and safe atmosphere in their interactions with their children, and promote the formation of safety attachment, which has a significant impact on the development of a healthy personality in children.

### Mediating role of core self-evaluation

This study found that core self-evaluation partially mediated the effect of adult attachment on the love psychological stress among college students. This indicates that adult attachment can directly affect the love psychological stress of college students, and can also affect the love psychological stress by their core self-evaluation. Hypothesis 2 is thus validated. Due to different upbringing experiences and personality characteristics, individuals respond differently to the same stimuli. On the basis of attachment relationships, children develop an internal work pattern or cognitive representation of interpersonal relationships. This model not only guides children’s expectations of their partners, but also their views on their own relationships with others [11]. Individuals with insecure attachment have positive representations of others, believing that others are good, but have negative self-representations, always feeling that they are not good enough or inferior to others, making it easier for them to develop lower self-evaluation. Individuals with low self-evaluation are filled with feelings of low self-worth and guilt, and they excessively need to validate and maintain their identity and value in the eyes of important others [33]. Additionally, individuals with low self-evaluation are more prone to strong feelings of depression, especially when faced with life events that undermine their self-defense or personal achievements, often accompanied by suicidal impulses [34]. Due to feelings of low self-worth and the fear of threats from others, individuals with low self-evaluation are more likely to experience greater psychological stress in love relationships and exhibit greater stress responses. This suggests that both parents and schools need to pay attention to educational methods, cultivate the ability of college students to live independently and take responsibility, trust and encourage them, and enable them to learn self-motivation and self-appreciation. This is crucial for the establishment of self-confidence, the development of independent ability, and the healthy development of personality in college students.

### Moderating effect of meaning in life

This study further reveals the important role of individual differences (meaning in life) in perceiving and regulating love psychological stress. Firstly, meaning in life negatively predicts love psychological stress. The stronger meaning in life, the less the love psychological stress, which is consistent with previous research findings [18]. This may be because college students with low meaning in life often have lower levels of psychological resilience, strong feelings of insecurity and fatigue, forcing them to adopt more negative, passive behavioral strategies to cope with difficulties and challenges [35]. Therefore, they usually find it difficult to actively build a supportive relationship network in love relationships, are unable to receive timely and effective help from others and society, thus increasing love psychological stress. Secondly, this study also found that meaning in life moderated the mediating effect of adult attachment on the love psychological stress through core self-evaluation in the latter half. That is, as the meaning in life of college students increases, the higher the level of core self-evaluation, the greater their love psychological stress. This result supports hypothesis *H*_3c_ and validates the viewpoint of the target process theory. Although core self-evaluation can reduce love psychological stress, college students with high meaning in life experience lower love psychological stress at the same level of core self-evaluation. Meaning in life as a positive psychological resource helps promote the healthy development of individuals both physically and mentally [36], such as core self-evaluation, thus helping individuals alleviate love psychological stress. At the same level of perceived core self-evaluation, individuals with low meaning in life tend to give up effort and experience more depression and anxiety when facing pressure in love relationships. This is also in line with the view of Das [37], which believes that meaning in life has several important functions for humans, including providing individuals with values and standards for judging behavior, as well as providing a sense of control over life events and self-worth. College students tend to feel confused in the early stages of adulthood, and helping them establish a sense of life meaning is a worthwhile educational task. College educators can help improve college students’ sense of life meaning through psychological interventions or other educational means, which will help them maintain a positive core self-evaluation and secure attachment pattern in stressful environments, ultimately fostering physical and mental health development.

Meaning in life did not exhibit a moderating effect on the direct path and the first half of the mediating path. Hypotheses 3a and 3b were not confirmed, indicating that the predictive role of adult attachment on college students’ love psychological stress and core self-evaluation was relatively stable. According to the cognitive appraisal theory of stress and the internal working model of attachment, individuals with insecure attachment are more inclined to close off their emotions, communicate less with others, and are more likely to experience negative emotions, negate self-worth, leading to low self-esteem and love psychological stress [4, 14]. Therefore, regardless of whether it is for college students with a high level of meaning in life or those with a low level of meaning in life, adult attachment can significantly affect college students’ love psychological stress and core self-evaluation.

### Theoretical and practical implications

Theoretically, this study is the first to comprehensively explore the combined effects of adult attachment, core self-evaluation, and meaning in life on the love psychological stress of college students, revealing the intrinsic correlation and regulatory mechanisms between adult attachment and factors of psychological stress. The research findings provide theoretical guidance for alleviating psychological stress in college students’ love relationships, and also expand the application of the cognitive appraisal theory of stress and the goal progress theory in the field of love psychological stress among college students. In terms of practical significance, firstly, by examining the mediating role of core self-evaluation and the moderating role of meaning in life, this study enriches previous research and deepens the understanding of the mechanism by which adult attachment affects the love psychological stress of college students. Adult attachment is related to physical and mental health development, and the love psychological stress among college students is a common psychological issue. Therefore, it is necessary for schools and families to raise awareness of this issue. Secondly, schools and families should help college students establish a correct view of love, and combine it with their own developmental characteristics to provide psychological education on love, in order to prevent and intervene in their love psychological stress. Finally, based on the roles of core self-evaluation and meaning in life, it may be considered to use group psychological counseling or other educational means to help college students shape a positive core self-evaluation, or to enhance college students’ meaning in life through career planning consultations and other means, in order to promote the healthy development of adult attachment patterns and ultimately sustain their physical and mental health development.

### Limitations and future directions

In addition to constructing a theoretical model and providing guidance, this study has limitations and provides directions for the future. Firstly, in terms of research methodology, this study is a cross-sectional study, making it difficult to reveal causal relationships between variables. Future research can use aggregated cross-sectional tracking designs to further verify causal relationships between variables. Secondly, in terms of research content, this study only explored the relationship between adult attachment and college students’ romantic stress, as well as cognitive mediating factors. For college students, the field of study is also very important, and related emotional factors (such as anxiety, depression, etc.) may also play a mediating role. Subsequent research could consider factors related to learning and emotions. Thirdly, in terms of moderating effects, this study only explored individual cognitive factors, but potential moderating factors related to genetics and environment should be emphasized in future research to build a more comprehensive model to systematically explain the formation and development process of adult attachment in college students. Finally, in terms of research subjects, a more diverse range of subjects (such as cross-regional, cross-cultural, etc.) should be included in subsequent research to enhance the universality of the findings.

## Conclusions

This study constructs a moderated mediation model, adult attachment is a risk factor for psychological stress in love among college students, with core self-evaluation and meaning in life playing mediating and moderating roles, respectively. The results of the study initially responded to the question of the significantly positive effect of adult attachment on love psychological stress in college students. Then, the mediating role of core self-evaluation was identified. Finally, this study clarified the extent to which meaning in life makes the effect of core self-evaluation on love psychological stress in college students significant. The psychological adjustment ability of students plays an essential role in their perception of personal competence. In addition, appropriate group psychological counseling and career planning counseling for different groups of students to equip them with high meaning in life to cope with social and external changes can help stimulate students’ core self-evaluation, thus alleviating love psychological stress of college students.

## Data Availability

Data and materials are available on request from the corresponding author.
